# Fentanyl Infusion Is Effective in the Treatment of Dyspnea in End-Stage Heart Failure With Renal Failure: A Case Report

**DOI:** 10.7759/cureus.12673

**Published:** 2021-01-13

**Authors:** Ryo Sakamoto, Atsuko Koyama

**Affiliations:** 1 Psychosomatic Medicine, Kindai University Faculty of Medicine, Osakasayama City, JPN

**Keywords:** dyspnea, end-stage heart failure, palliative care, fentanyl

## Abstract

Patients with end-stage heart failure may require hospitalization for the treatment of respiratory distress. Morphine may be used to relieve symptoms. However, use of morphine is problematic because heart failure is often associated with renal dysfunction. In this case report, intravenous fentanyl infusion reduced dyspnea in a patient with end-stage heart failure who was on dialysis induction to treat renal failure. The patient was able to eat and sleep after administration of an intravenous fentanyl solution and experienced no apparent respiratory depression as a side effect of fentanyl. There have been few reports of dyspnea relief with fentanyl infusion. Because of its adjustable duration of effect, intravenous fentanyl may be a more effective and useful option than rapid or extended-release agents in cases such as this one.

## Introduction

Breathlessness is distressing in palliative patients with end-stage heart failure. Many patients are admitted to the hospital for relief of this symptom [1]. Breathlessness associated with end-stage heart failure is intractable, and there is no effective treatment for this condition. Strong opioids (e.g., morphine) are the only drugs with evidence for symptomatic treatment of shortness of breath. In most cases, oral or parenteral morphine is administered [2]. However, morphine is used with caution in patients with impaired kidney function, and heart failure is often associated with kidney damage. Heart failure is common among patients with renal failure (present in 63% of cases), and 29% of patients with heart failure have concomitant moderate-to-severe renal impairment [3]. Strong opioids that can be used for respiratory distress in heart failure include hydromorphone and oxycodone in addition to morphine. However, these drugs should be administered with caution in the presence of renal dysfunction. Among opioids, fentanyl can be used without problems in patients with renal impairment. Intranasal fentanyl has been reported to be effective for breathlessness in heart failure [1]. However, there are few reports on use of the injectable formulation for this indication. Fentanyl has the side effect of respiratory depression, which raises concern about the difficulty of adjusting the dosage with continuous intravenous infusion. The problem with fentanyl is that it is much more potent than morphine and has a much faster onset of action. Therefore, the management of opioid infusions needs to be much more intensive than when administering intermittent opioids. Although this should be handled with care, continuous intravenous infusion is expected to have a sustained effect and may have a positive impact on nighttime sleep disturbances associated with dyspnea. In this article, we report the case of a patient with end-stage heart failure on hemodialysis for renal failure who responded to continuous intravenous fentanyl infusion and discuss the aspects of this treatment.

## Case presentation

**Figure 1:**
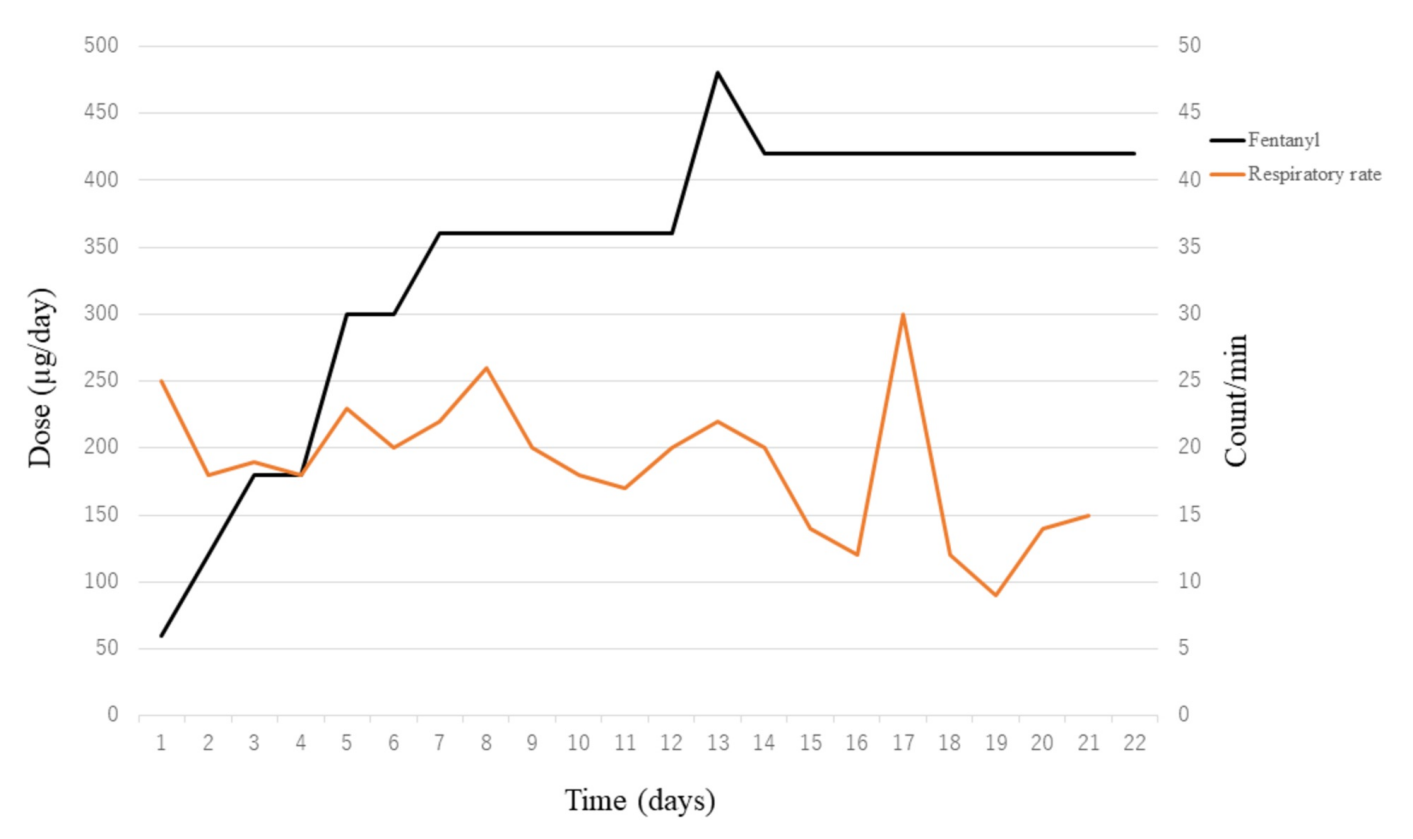
Daily dose of fentanyl injection and respiratory rate.

An 83-year-old man was admitted to our Cardiology Department for treatment of chronic heart failure caused by dilated cardiomyopathy. The patient also had chronic renal failure associated with heart failure and received dialysis three times per week. After admission, the patient’s heart disease was treated aggressively, but his doctors found it difficult to treat him further. Therefore, the patient accepted to be changed to palliative treatment and referred to the palliative care team (PCT) for relief of dyspnea. At the patient’s first PCT visit, he was given 5 L/min of oxygen and had an oxygen saturation of 91%. However, he was aware of dyspnea, was unable to eat or drink, and was unable to sleep well at night. The PCT and the attending physician discussed a treatment plan for dyspnea; because of the complication of renal failure, intravenous fentanyl infusion was started at 2.5 µg/h instead of morphine. No bradypnea was observed during administration of fentanyl, and the patient’s respiratory rate remained 20 breaths/min. However, because there was no change in subjective symptoms of dyspnea, the dose was increased to 5 µg/h the next day and to 7.5 µg/h the day after that. On the fourth day of the PCT intervention, the patient reported, “I feel more comfortable with my body now than at any time during this hospital stay. I was able to eat one piece of bread for breakfast and a few side dishes.” However, because of the appearance of respiratory distress after hemodialysis, the dose of fentanyl was increased to 10 µg/h. When the PCT visited the patient’s room during the fifth day of the intervention, he remarked, “I slept well last night. I slept for five hours.” However, because of persistent complaints of nighttime dyspnea, the dose of fentanyl was increased each day (to 12.5 µg/h and then to 15 µg/h). During this period, the oxygen flow rate increased from 5 L/min to 8 L/min, which may have been influenced by worsening of the primary disease. Thereafter, the patient’s symptoms remained unchanged for four days. However, on the 11th day, the oxygen flow rate was increased to 10 L/min, yet the patient had difficulty with oral intake and continued to have difficulty breathing at night; hence, midazolam was started intermittently at night only. On the afternoon of the 12th day, dyspnea recurred and the dose of fentanyl was increased to 17.5 µg/h. Dyspnea continued on day 13 and increasing the fentanyl dose to 20 µg/h did not improve symptoms; therefore, midazolam was started during the day to target “light sedation” on the Richmond Agitation-Sedation Scale. On day 14 of the intervention, the subjective symptoms of dyspnea improved, and sleep and food intake increased. Therefore, the dose of fentanyl was reduced to 17.5 µg/h. The course of the fentanyl dose in this case is shown in Figure 1.

There was no apparent bradypnea as a side effect when fentanyl was gradually increased. Dialysis, which was painful for the patient, was reduced to once per week at his request. The patient’s activity gradually decreased and he died on the 22nd day.

## Discussion

The most important finding in this case was the efficacy of continuous intravenous fentanyl in the treatment of dyspnea due to chronic heart failure. In particular, the continuous administration of fentanyl improved dyspnea at night. In addition to the injectable form of fentanyl, sublingual tablets, buccal tablets, and extended-release patches have been approved in Japan. In high-risk patients such as this case, initial titration can be recommended initially and then consideration of conversion to the transdermal route. Rapid-onset opioid (ROO) preparations, such as sublingual and buccal tablets, have a shorter time to maximum effect and duration of effect, whereas sustained-release patches have a longer maximum time and duration of effect [4,5] (Table 1).

**Table 1:** Pharmacokinetics and clinical parameters of fentanyl in Japan.

Parameter	Sublingual tablet	Buccal tablet	Sustained-release patch	Intravenous injection
Product and fentanyl dosages (µg)	Abstral® (100, 200, 400,)	E-fen® (50, 100, 200, 400, 600, 800)	Durotep® (12.5, 25, 50, 75, 100 µg/h over 72 h)	Fentanyl (100, 250,500)
Onset of action	5-15 min	5-15 min	6 h	Almost immediate
Duration	60 min	At least 60 min	Up to 12 h after patch removal	0.5-1 h
Half-life elimination	3-14 h	3-12 h	20-27 h	2-4 h
Time to peak	20-480 min (median: 20-40 min)	20-240 min (median: 47 min)	20-72 h	-

Thus, the former is not ideal for use before bedtime because its effect does not last until morning, whereas the latter has a longer half-life, making it difficult to adjust the dose if side effects such as respiratory depression appear. One disadvantage of the injectable formulation is the increased risk of delirium associated with the intravenous indwelling [6]. One option for sleep support is combining psychotropic medications with ROO preparations. However, antidepressants and antipsychotics have been reported to pose risks in patients with chronic heart failure [7,8]. Benzodiazepines also pose a potential risk of delirium [9], although they have not been shown to pose a clear risk for heart failure [6]. It is unclear how ramelteon and suvorexant affect chronic heart failure, but they may be worth trying if they can be taken orally. However, there have been no reports of these drugs relieving dyspnea symptoms in patients with heart failure. Another important point is that the results suggest that the effective dose of fentanyl injection for dyspnea may have to be higher than the effective dose of morphine. In the present case, the fentanyl dose was started at 2.5 µg/h and increased to 20 µg/h over a 13-day period. This amount corresponds to 1 mg/h of injectable morphine. In general, 7.5 to 15 mg of immediate-release morphine is reported to be effective for dyspnea in chronic heart failure [10]. The sustained-release morphine is reported to be effective at 10 mg/day [11]. The result is about 0.2 mg/h in an injectable formulation of morphine. In our patient, fentanyl did not cause any noticeable side effects such as nausea and constipation, and the drug may be used without problems if the dose is increased with careful monitoring of vital signs. This case suggests that fentanyl may be a treatment option for symptoms of dyspnea resulting from chronic heart failure. Given its effectiveness in the treatment of dyspnea associated with chronic heart failure, its use for this indication is appropriate.

## Conclusions

In this case, fentanyl injection was shown to be a useful modality for symptom relief in a patient who presented with dyspnea symptoms of chronic heart failure. Therefore, fentanyl is considered a potential future treatment option.
